# Probiotics for inflammatory bowel disease: Is there sufficient evidence?

**DOI:** 10.1515/biol-2022-0821

**Published:** 2024-04-05

**Authors:** Yueying Ma, Dandan Yang, Jin Huang, Kunli Liu, Huirong Liu, Huangan Wu, Chunhui Bao

**Affiliations:** Yueyang Hospital of Integrated Chinese and Western Medicine, Shanghai University of Traditional Chinese Medicine, Shanghai 200437, China; Shanghai University of Traditional Chinese Medicine, Shanghai 201203, China; Hong Kong Baptist University, Hong Kong 999077, China; Key Laboratory of Acupuncture and Immunological Effects, Shanghai University of Traditional Chinese Medicine, Shanghai 200030, China

**Keywords:** probiotics, inflammatory bowel disease, ulcerative colitis, Crohn’s disease, intestinal microbiome, gut health

## Abstract

Inflammatory bowel disease (IBD) refers to chronic inflammatory disorders of the gut. Ulcerative colitis (UC) and Crohn’s disease (CD) are two subtypes of IBD. Evidence suggests that the intestinal microbiota plays a role in the pathogenesis of IBD, so probiotics have garnered a lot of interest as a potential treatment or prevention for IBD. However, clinical evidence of the efficacy of probiotics is still debatable. We performed a literature review. An advanced search considered clinical studies on probiotic for IBD from inception to 2023 in PubMed, Embase, Cochrane Library, and Web of Science. In the treatment of UC with probiotics, only *Escherichia coli* Nissle 1917 for maintenance treatment of UC in remission, and *Bifidobacterium* and VSL#3 for induction of remission in patients with mild to moderately active UC have shown strong evidence. Currently, there are no definitive conclusions regarding the effectiveness of probiotics in CD. The mechanism of probiotic treatment for IBD may be related to reducing oxidative stress, repairing the intestinal barrier, regulating intestinal flora balance, and modulating intestinal immune response. Differences in the benefits of probiotics between CD and UC may be attributable to the different lesion extent and immune-mediated pathophysiology. More robust randomized clinical trials are required to validate the efficacy and safety of diverse probiotic strains in IBD.

## 1 Introduction

Inflammatory bowel disease (IBD), including ulcerative colitis (UC) and Crohn’s disease (CD), is a chronic inflammatory disorder of the gastrointestinal tract with a disease course characterized by frequent relapses. The clinical manifestations of IBD include (hemorrhagic) diarrhea, abdominal pain, weight loss and/or fatigue. Some patients also exhibit extra-intestinal manifestations such as skin lesions, pulmonary symptoms, or arthritis [1,2].

Initially, IBD was perceived as a condition predominantly prevalent in developed regions. However, an increasing number of cases have been observed across the world and the prevalence rates have shown an increasing trend [3]. The precise etiology of IBD is not well characterized. Host genetics, luminal environment, and the external environment have all been implicated in its causation [4,5,6]. A wide body of evidence from clinical and experimental studies suggests that dysbiosis of the intestinal bacteria characterized by structural and functional alterations of the gut microbiome may contribute to the development of IBD [7]. Some studies have shown that the emerging pathogenic bacteria may lead to increased incidence and severity of IBD in genetically susceptible individuals [8]. Most of the human IBD microbiome research conducted so far has focused on microbial composition rather than function [9]. Increased inflammation may be caused by the presence of bacteria which are not normally resident on the mucosal surface [10]. Owing to the presumed cause-and-effect relation between gut microbial dysbiosis and the development of IBD, various microbial-based therapies, such as probiotics [3], prebiotics [11], fecal microbiota transplantation [12], and nutritional supplements [13], are now available for the treatment of this disease.

Probiotics are defined as live microorganisms that confer a health benefit to the host by favorably altering the gut microflora after oral intake in adequate amounts [14]. Owing to their ease of administration and low cost, probiotics stand out among the many treatment therapies. They are often the most-used additional therapy in gastrointestinal diseases and are frequently recommended by physicians [15,16]. Microorganisms must meet several criteria to qualify as probiotic. They should be identified at the level of genus, species, and strain and should have demonstrable safety for clinical use, and the ability to survive intestinal transit. Most importantly, their clinical health effects should be validated in at least one phase 2 study [17]. However, although probiotics are widely used and generally regarded as safe [18], there is no clear consensus on the efficacy and safety of probiotic use in IBD. According to a meta-analysis, IBD patients receiving probiotics showed a higher risk of total adverse effects and gastrointestinal symptoms than those taking placebo; however, only abdominal pain showed statistically significant difference [19].

The strategy for manipulation of the microbial composition targeting the gut microbiota in IBD has been a research hotspot in recent decades. Each probiotic strain may have different immunoregulatory properties, and probiotics can modulate the intestinal immune response indirectly or directly [20]. Studies have shown that it is possible to modify the intestinal environment of patients with IBD by oral intake of probiotics [21,22].

## 2 Methodology

Owing to the conflicting evidence regarding the effectiveness of probiotics for IBD and the usage of several different kinds of probiotics, we conducted a literature review to summarize the results and conclusions of the clinical trials of probiotics in IBD. Advanced searches were performed in PubMed, Embase, Cochrane Library, and Web of Science. The search was performed by applying the following keywords alone or in combination: “Probiotic,” “*Bifidobacterium*,” “*Escherichia coli* Nissle 1917,” “*Lactobacillus*,” “Bifid Triple Viable,” “*Saccharomyces*,” “Inflammatory bowel disease,” “ulcerative colitis,” and “Crohn’s disease.” We considered clinical studies on probiotics for IBD from inception to 2023. A total of 55 articles were included. In particular, we sought to clarify the role of probiotics in the induction or maintenance of remission of IBD. In addition, we explored the potential mechanisms of the role of probiotics. This review may help inform the clinical use of probiotics in IBD.

## 3 Probiotics in the treatment of IBD

The clinical course of IBD is characterized by episodes of exacerbation and remission. Current treatment strategies for this disease include induction of remission first, followed by possibly long-term maintenance of remission [23]. We reviewed a total of 53 clinical trials of oral probiotics including clinical trials in IBD conducted over the last two decades. We organized these studies around the different subtypes of IBD (UC or CD) and the different disease stages (active or remission). The purpose of this review is to provide a broad overview of the studies on probiotics and their impact on IBD, as well as to discuss the effects of different probiotic strains.

### 3.1 Probiotics in UC

#### 3.1.1 Induction of remission in active UC

Studies that investigated the induction of remission in active UC and evaluated the clinical outcomes are summarized in Table 1.

**Table 1:** Probiotics in UC for the induction of remission

Probiotic	Author/year of publication/country	Study design	Methods	Results	Summary
Bifidobacteria-fermented milk	Kato et al. 2004 [24]	RPCT	**Patients:** mild to moderate active UC	Decrease in the CAI; reduce in the endoscopic activity index and histological score; increase in fecal butyrate, propionate, and short-chain fatty acid concentrations	A beneficial effect was observed
			**Treatment period:** 12 weeks		
			**T(** ***n*** **= 10):** bifidobacteria-fermented milk, 100 mL/day; conventional treatment (sulfasalazine or mesalazine)		
	Japan				
			**C(** ***n*** **= 10):** placebo; conventional treatment (sulfasalazine or mesalazine)		
Bifidobacteria-fermented milk	Ishikawa et al. 2011 [25]	OL	**Patients:** mild to moderate active UC	Decrease in the endoscopic score, the MPO amounts, the fecal number of Bacteroidaceae, and the fecal pH	A beneficial effect was observed
			**Treatment period:** 1 year		
			**T(** ***n*** **= 21):** 1 g of the freeze-dried powder containing probiotic *B. breve* strain Yakult (10^9^ CFU/g) thrice a day and 5.5 g of galacto-oligosac charide once a day		
	Japan				
			**C(** ***n*** **= 20):** none		
*B. infantis* 35624	Groeger et al. 2013 [29]	DB, RPCT	**Patients:** mild to moderate active UC	Reduction in the levels of CRP and TNF-α	A beneficial effect was observed
			**Treatment period:** 6 weeks		
			**T(** ***n*** **= 13):** sachets containing 1 × 10^10^ CFU viable *B. infantis* 35264		
	Ireland				
			**C(** ***n*** **= 9):** placebo		
*B. longum* 536	Tamaki et al. 2016 [27]	DB, RPCT	**Patients:** mild-to-moderate active UC	Reduction in UCDAI scores, EI and Mayo subscores, and rectal bleeding; clinical remission	A beneficial effect was observed
			**Treatment period:** 8 weeks		
			**T(** ***n*** **= 28):** 2–3 × 10^11^ freeze-dried viable *B. longum* 5,363 times daily		
	Japan				
			**C(** ***n*** **= 28):** placebo		
*B. longum*	Furrie 2005 [28]	DB, RPCT	**Patients:** active UC	Reduce in sigmoidoscopy scores, mRNA levels for human beta defensins 2, 3, and 4, TNF-α, and IL-1α	A beneficial effect was observed
			**Treatment period:** 4 weeks		
			**T(** ***n*** **= 9):** 2 × 10^11^ freeze-dried viable *B. longum* in a gelatin capsule and a sachet containing 6 g of prebiotic fructo-oligosaccharide/inulin mix		
	United Kingdom				
			**C(** ***n*** **= 9):** placebo		
*B. longum*	Takeda et al. 2009 [30]	OL	**Patients:** mild to moderate active UC	Decrease in the mean CAI at 8, 12, and 24 weeks	A beneficial effect was observed
			**Treatment period:** 24 weeks		
			**T(** ***n*** **= 14):** 2–3 × 10^11^ freeze-dried viable *B. longum*		
			**No control group**		
	Japan				
*Bifidobacterium*	Nagasaki et al. 2010 [26]	Case report	**Patients:** active UC	Improve in physical condition and colonoscopic findings; possible to reduce the steroid dose without relapse	A beneficial effect was observed
			**Treatment period:** 1 week		
			**Case Report(** ***n*** **= 1):** *Bifidobacterium* 6 mg/day		
	Japan				
*E. coli* Nissle 1917	Petersen et al. 2014 [32]	DB, RPCT	**Patients:** active UC	No support for the use of *E. coli* Nissle as an add-on treatment to conventional therapies for active UC colitis	No benefit was observed
			**Treatment period:** 7 weeks		
			**T1(** ***n*** **= 25):** ciprofloxacin (500 mg twice daily) for 1 week followed by *E. coli* Nissle 1917 for 7 weeks (100 mg × 1 for 4 days followed by 100 mg × 2 daily for the rest of the period)		
	Denmark				
			**T2(** ***n*** **= 25):** ciprofloxacin for 1 week followed by placebo for 7 weeks		
			**T3(** ***n*** **= 25):** placebo for 1 week followed by *E. coli* Nissle 1917 for 7 weeks		
			**T4(** ***n*** **= 25):** placebo for 1 week followed by placebo for 7 weeks		
*E. coli* Nissle 1917	Park et al. 2022 [34]	DB, RCT	**Patients:** using 5-ASA and presenting mild-to-moderate active UC	Safe and effective in preventing the exacerbation of IBDQ scores and achieving clinical responses and endoscopic remission in patients with mild-to-moderate UC	A beneficial effect was observed
			**Treatment period:** a maximum of 12 months		
			**T(** ***n*** **= 67):** *E. coli* Nissle 1917 (2.5 × 10^9^ CFU per capsule) one capsule/day from day 1 to day 4 and two capsules/day from day 5 for 8 weeks		
	Korea		**C(** ***n*** **= 66):** placebo		
*E coli* Nissle 1917	Rembacken et al. 1999 [33]	DB, DD, RCT	**Patients:** mild, moderate, and severe active UC	An equivalent effect to mesalazine in inducing remission of UC	A beneficial effect was observed
			**Treatment period:** a maximum of 12 months		
			**T(** ***n*** **= 57):** gentamicin 80 mg three times daily for 1 week followed by *E. coli* Nissle 1917 at a dose of two capsules twice daily (2.5 × 10^10^ viable bacteria per capsule) for 12 weeks		
	United Kingdom				
			**C(** ***n*** **= 59):** gentamicin 80 mg for 1 week followed by mesalazine 800 mg three times daily		
*Lactobacillus delbruekii*, *L. fermentum*	Hegazy and El-Bedewy 2010 [35]	RCT	**Patients:** mild-to-moderate UC	Decrease the colonic concentration of IL-6, expression of TNF-α and NF-κB p65, leukocyte recruitment (demonstrated by a decrease in colonic MPO activity), and the level of fecal calprotectin	A beneficial effect was observed
			**Treatment period:** 8 weeks		
			**T(** ***n*** **= 15):** 10^10^ CFU/day; sulfasalazine 2,400 mg/day		
			**C(** ***n*** **= 15):** sulfasalazine 2,400 mg/day		
	Egypt				
*L. casei* DG	D’Inca et al. 2011 [37]	RCT	**Patients:** mild left-sided UC	Eight weeks of oral mesalazine did not provoke significant changes in the mucosa-associated microbiota in UC patients, nor did have a significant effect on the counts of *Enterobacteriaceae* spp. or of *Lactobacillus* spp	No benefit was observed
			**Treatment period:** 8 weeks		
			**T(** ***n*** **= 8):** oral mesalazine (2.4 g/day) plus oral *L. casei* DG (8 × 10^8^ CFU) twice daily		
	Italy				
			**C(** ***n*** **= 7):** oral mesalazine (2.4 g/day)		
*L. reuteri* ATCC 55730	Oliva et al. 2012 [36]	RPCT	**Patients:** mild-to-moderate distal UC**Treatment period:** 8 weeks	Decrease in the Mayo score and the behavior of the histological score	A beneficial effect was observed
			**T(** ***n*** **= 20):** before bedtime an enema solution containing 10^10^ CFU of *L. reuteri* ATCC 55730		
	Italy				
			**C(** ***n*** **= 20):** placebo		
*L. rhamnosus* GG, ATCC 5 03	Meini et al. 2015 [38]	Case report	**Patients:** active UC	Bacteremia	An adverse event was observed
			**Treatment period:** 13 days		
			**Case Report(** ***n*** **= 1):** *L. rhamnosus* GG 6 × 10^9^ CFU once daily; corticosteroids and mesalazine		
	Italy				
*Lactobacillus* and *Bifidobacterium*	Agraib et al. 2022 [39]	DB, RPCT	**Patients:** mild-to-moderate active UC	Improvement in the Partial Mayo score, stool frequency, global assessment, and total PMS score; reduction in CRP and IgA level; increase in hemoglobin, hematocrit, RBC levels, and IL-10 levels	A beneficial effect was observed
			**Treatment period:** 6 weeks		
			**T(** ***n*** **= 15):** 3 × 10^10^ of probiotic product “probiotic 10 billion active cells^®^” (Jamieson Laboratories, Canada N8W5B5) capsules (containing nine *Lactobacillus* and five *Bifidobacterium* species) daily		
	Jordan				
			**C(** ***n*** **= 15):** placebo		
Canada N8W5B5	Rayyan et al. 2023 [40]	DB, RPCT	**Patients:** mild-to-moderate UC	Improvement in the scores of the systemic domain, social domain, bowel domain, emotional domain, and total SIBDQ	A beneficial effect was observed
			**Treatment period:** 6 weeks		
			**T(** ***n*** **= 16):** 3 × 10^10^ probiotic capsules containing nine *Lactobacillus* and five *Bifidobacterium* species 3 times daily		
			**C(** ***n*** **= 14):** placebo		
	Jordan				
*Lactobacillus salivarius*, *L. acidophilus*, and *Bifidobacterium bifidus* strain BGN4	Palumbo et al. 2016 [41]	RCT	**Patients:** moderate-to-severe UC	Ameliorate the clinical response; shorten the time of recovery; improve the stool frequency and intestinal mucosa aspect in the endoscopic picture	A beneficial effect was observed
			**Treatment period:** 2 years		
			**T(** ***n*** **= 30):** a double daily administration of a probiotic; mesalazine 1,200 mg		
			**C(** ***n*** **= 30):** mesalazine 1,200 mg		
	Italy				
VSL#3	Tursi et al. 2004 [42]	RCT	**Patients:** mild-to-moderate UC	The balsalazide/VSL#3 combination was faster in obtaining remission; better in improving well-being, bowel frequency, endoscopic, and histological scores	A beneficial effect was observed
			**Treatment period:** 8 weeks		
			**T(** ***n*** **= 30):** 2.25 g balsalazide daily as capsules containing 750 mg of balsalazide disodium, plus 3 g VSL#3 daily as 1 g bags containing 3 × 10^11^ viable lyophilized bacteria per gram		
	Italy				
			**C1(** ***n*** **= 30):** 4.50 g balsalazide		
			**C1(** ***n*** **= 30):** 2.4 g mesalazine		
VSL#3	Miele et al. 2009 [46]	DB, RPCT	**Patients:** mild-to-moderate active UC; children age between 1.7 and 16.1	Maintained remission; decrease in endoscopic and histological scores	A beneficial effect was observed
			**Treatment period:** 1 year		
	Italy		**T(** ***n*** **= 14):** VSL#3 4.5 × 10^11^ – 1.8 × 10^12^ (based on weight) bacteria/day; concomitant steroid induction and mesalamine		
			**C(** ***n*** **= 15):** placebo; mesalazine		
VSL#3	Sood et al. 2009 [43]	DB, RPCT	**Patients:** mild-to-moderate UC	Decrease in UCDAI scores; improvement in stool frequency score, blood in the stool score, mucosal appearance, and physician’s global assessment	A beneficial effect was observed
			**Treatment period:** 12 weeks		
			**T(** ***n*** **= 77):** 3.6 × 10^12^ CFU VSL#3 twice daily		
			**C(** ***n*** **= 70):** placebo		
	India				
VSL#3	Tursi et al. 2010 [44]	DB, RPCT	**Patients:** mild-to-moderate UC	Decrease in UCDAI scores; reduction in rectal bleeding	A beneficial effect was observed
			**Treatment period:** 8 weeks		
			**T(** ***n*** **= 71):** VSL#3 twice daily at a dose of 3.6 × 10^12^ CFU/day		
	Italy				
			**C(** ***n*** **= 73):** placebo		
VSL#3	Ng et al. 2010 [45]	DB, RPCT	**Patients:** mild-to-moderately active UC	Increase in IL-10 and IL-12p40; decrease in DC TLR-2 expression and IL-12p40 production	A beneficial effect was observed
			**Treatment period:** 8 weeks		
			**T(** ***n*** **= 14):** 2 sachets twice/day of VSL#3 (3.6 × 10^12^ bacteria)		
	United Kingdom				
			**C(** ***n*** **= 14):** placebo		
VSL#3	Huynh et al. 2009 [47]	OL	**Patients:** children age between 3 and 17; mild to moderate acute exacerbation UC; had a duration of exacerbated symptoms lasting less than 4 weeks	Decrease in SCCAI; improvement in the mean Mayo endoscopic score, TNF-α, IFN-γ, CRP, and ESR	A beneficial effect was observed
			**Treatment period:** 8 weeks		
	Canada				
			**T(** ***n*** **= 18):** a dose of probiotic based on their age (from one-half sachet to two & one-half sachets twice daily		
			**No control group**		
VSL#3	Bibiloni et al. 2005 [48]	OL	**Patients:** mild-to-moderate active UC	Decrease in UCDAI in patients entering remission or responding; clinical remission	A beneficial effect was observed
			**Treatment period:** 6 weeks		
			**T(** ***n*** **= 34):** VSL#3 (3.6 × 10^12^ bacteria) daily in two divided doses		
	Canada, Italy, USA				
			**No control group**		
VSL#3	Soo et al. 2008 [49]	OL	**Patients:** mild-to-moderate active or quiescent UC colitis	Decrease in UCDAI; increase in mucosal alkaline sphingomyelinase activity	A beneficial effect was observed
			**Treatment period:** 5 weeks		
	Canada		**T(** ***n*** **= 15):** one sachet of VSL#3 (containing 9 × 10^11^ lyophilized bacteria) orally two times per day		
			**No control group**		
BIO-THREE	Tsuda et al. 2007 [51]	OL	**Patients:** mild-to-moderate distal UC	Decrease in UCDAI score in patients who achieved either remission or response; clinical and endoscopic improvement accompanying changes of the intestinal microflora pattern	A beneficial effect was observed
			**Treatment period:** 4 weeks		
			**T(** ***n*** **= 20):** BIO-THREE tablets (2 mg *S. faecalis* T-110, 10 mg *C. butyricum* TO-A and 10 mg *B. mesentericus* TO-A) per day		
			**No control group**		
	Japan				
Bifid Triple Viable	Li et al. 2012 [52]	RCT	**Patients:** mild-to-moderate active UC	Decrease in the clinical symptom score, colon mucosa inflammation score, and IL-1β expression; increase in IL-10 and IgA expressions in colon mucosa and the ratio of peripheral blood CD4+ T cell to CD8+ T cell	A beneficial effect was observed
			**Treatment period:** 2 months		
			**T(** ***n*** **= 41):** Bifid Triple Viable 2 capsules three times daily and mesalazine 1 g two times daily		
	China				
			**C(** ***n*** **= 41):** mesalazine		
Bifid Triple Viable	Cui et al. 2004 [53]	RPCT	**Patients:** active UC	Increase in the concentration of fecal lactobacilli and bifidobacterial; improvement in the activation of NF-κB and the expression of IL-10; decrease in the expressions of TNF-α and IL-1β; helpful in maintaining remission and preventing relapse of UC	A beneficial effect was observed
			**Treatment period:** 8 weeks		
			**T(** ***n*** **= 30):** BIFICO 1.26 g/day		
			**C(** ***n*** **= 30):** placebo		
	China				
*S. boulardii*	Guslandi et al. 2003 [54]	OL	**Patients:** mild to moderate clinical flare-up of UC	Reduction in the clinical index score; clinical remission confirmed by sigmoidoscopy	A beneficial effect was observed
			**Treatment period:** 4 weeks		
			**T(** ***n*** **= 25):** *S. boulardii* 250 mg three times a day; mesalazine		
	Italy				
			**No control group**		

OL: open label; DB: double blind; DD: double dummy; RPCT: randomized placebo-controlled trial; RCT: randomized controlled trial; C: control group; T: treatment group.

##### 3.1.1.1 Bifidobacterium

For a long time, fermented dairy products have been utilized to treat gastrointestinal disorders. For the treatment of active UC, bifidobacteria-fermented milk containing live bifidobacteria, Yakult strains of *Bifidobacterium breve*, *Bifidobacterium bifidum*, and *Lactobacillus acidophilus* may be safe and more effective than conventional treatment (sulfasalazine or mesalazine) alone. In a randomized controlled trial (RCT) [24] of bifidobacteria-fermented milk, the probiotic group showed significantly lower clinical activity index (CAI) than the placebo group, as well as greater concentrations of fecal butyrate, propionate, and short-chain fatty acids. In an uncontrolled trial by Ishikawa et al. [25], bifidobacteria-fermented milk was found to be more effective than placebo in reducing fecal myeloperoxidase (MPO) levels, the number of bacillus-like bacteria, and fecal pH. Moreover, bifidobacteria-fermented milk was found to lower the endoscopic activity index in patients with active UC in both trials. Nagasaki et al. [26] reported a 71-year-old patient with active UC who was administered *Bifidobacteria* 1 week after the failure of several treatments and showed improvement in physical condition and colonoscopy findings, with the possibility of reducing the steroid dose without relapse. Three randomized placebo-controlled trials [27,28,29] also showed the effect of *Bifidobacterium* in inducing remission in patients with active UC. Administration of *Bifidobacterium longum* for 8 weeks [27] resulted in greater reduction in the UC disease activity index (UCDAI) scores, endoscopic index, Mayo subscores, and rectal bleeding, as well as induced clinical remission. Administration of *B. longum* for 4 weeks [28] resulted in better reduction in sigmoidoscopy scores, mRNA levels of human beta-defensins 2, 3, and 4, tumor necrosis factor-α (TNF-α), and interleukin (IL)-1α. Administration of *Bifidobacteria infantis* 35624 for 6 weeks [29] resulted in better reduction in the levels C-reactive protein (CRP) and TNF-α. In an uncontrolled study by Takeda et al. [30], administration of *B. longum* for 24 weeks decreased the mean CAI.

##### 3.1.1.2 *E. coli* Nissle 1917

*E. coli* Nissle 1917, a non-pathogenic strain of *E. coli*, is probably the most intensively investigated bacterial strain among the Gram-negative microorganisms [31]. Three RCTs that investigated the effect of *E. coli* Nissle 1917 in active UC yielded inconsistent results. Petersen et al. [32] found no benefit of use of *E. coli* Nissle 1917 as an add-on treatment with Ciprofloxacin for active UC. However, in a study of 116 patients with active UC conducted by Rembacken et al. [33], the effect of 1-week treatment with gentamicin followed by *E. coli* Nissle 1917 was equivalent to that of mesalazine in inducing UC remission. A study of 133 patients also reported the safety and efficacy of *E. coli* Nissle 1917 in preventing the exacerbation of IBDQ scores and achieving clinical responses and endoscopic remission in patients with mild-to-moderate UC [34]. The effect of *E. coli* Nissle 1917 on inducing remission in UC could not be clarified because the three studies used different drugs as the underlying treatment.

##### 3.1.1.3 Lactobacillus

Three RCTs have investigated the effect of *Lactobacillus* in UC. Hegazy and El-Bedewy [35] found that *Lactobacillus* not only decreased the colonic concentration of IL-6 and protein expressions of TNF-α and NF-κB p65 but also reduced the leukocyte recruitment (demonstrated by a decrease in colonic MPO activity) and the level of fecal calprotectin in patients with active UC. A decrease in the Mayo score and the behavior of the histological score were also found by Oliva et al. [36]. In contrast, D’Inca et al. [37] found no effect of 5-ASA coupled with oral *Lactobacillus casei DG* on the colonic flora and TLR expression, but when coupled with rectally administered *L. casei DG*, it modified colonic microbiota by increasing *Lactobacillus* spp. and mucosal IL-10, while reducing Enterobacteriaceae, TLR-4, and interleukin (IL)-1b mRNA levels. Meini et al. [38] documented a case of *Lactobacillus*-induced bacteremia after oral administration, which was resistant to vancomycin. A combination of *Lactobacillus* and *Bifidobacterium* was also shown to induce remission in UC patients [39] and improve the quality of life in mild to moderate UC patients [40]. A combination of *Lactobacillus* with other strains investigated by Palumbo et al. [41] reported a positive effect used in conjunction with mesalazine in moderate-to-severe active UC as demonstrated by achievement of clinical response, shortened time to recovery, and improved stool frequency and endoscopic intestinal mucosa scores. The efficacy of *Lactobacillus* for UC appears to be unclear and needs to be supported by more high-quality evidence.

##### 3.1.1.4 VSL#3

VSL#3 is very diverse probiotic containing multiple different strains of *Lactobacillus*, *Bifidobacterium*, and *Streptococcus* genera. There are eight trials, including six adult studies and two pediatric studies. All these studies showed a beneficial effect of VSL#3 in mild-to-moderate active UC. Tursi et al. [42] compared VSL#3 combined with low-dose balsalazide to medium-dose balsalazide or mesalazine alone and found that the combination helped achieve faster remission and better-improved well-being, bowel frequency, endoscopic, and histological scores. Treatment with VSL#3 in combination with commonly used anti-inflammatory drugs seems to be more effective than treatment with anti-inflammatory drugs alone. Four double-blind RCTs [43,44,45,46] were performed with VSL#3. The dose of 3.6 × 10^12^ VSL#3 CFU per day in adults was found to be sufficient to achieve a good clinical response. Two large studies [43,44] found greater decrease in UCDAI scores and significantly more patients with active UC improved at least 50% in CAI after VSL#3 treatment for 12 and 8 weeks than placebo, respectively. Similar results were also obtained in three open-label studies. Huynh et al. [47] found a decrease in simple clinical colitis activity index (SCCAI) and improvement in the mean Mayo endoscopic score, TNF-α, interferon-gamma (IFN-γ), CRP, and erythrocyte sedimentation rate (ESR). Bibiloni et al. [48] found a decrease in UCDAI. Soo et al. [49] found a decrease in UCDAI and an increase in mucosal alkaline sphingomyelinase activity. According to the 4th Yale/Harvard Workshop on Probiotic Recommendations in 2015 [50], VSL#3 was rated as grade B recommendations in inducing UC remission. None of the studies on VSL#3 found any side effects or ineffectiveness. These studies demonstrated the potential of VSL#3 as a stand-alone or additional treatment for active UC.

##### 3.1.1.5 Other probiotics

Several other probiotic stains have also been studied, including BIO-THREE, Bifid Triple Viable, and *Saccharomyces boulardii*. In the study by Tsuda et al. [51], BIO-THREE was found to decrease UCDAI score, alter the intestinal microflora pattern, and improve clinical and endoscopic conditions. Li et al. [52] found that Bifid Triple Viable administered with mesalazine was better than mesalazine alone in decreasing the clinical symptom score, colon mucosa inflammation score, and IL-1β expression and in increasing IL-10 and IgA expressions in colon mucosa and the ratio of peripheral blood CD4+ T cell to CD8+ T cell. An RCT of Bifid Triple Viable [53] showed similar results, which suggests that use of Bifid Triple Viable alone may also achieve the same effect. In an uncontrolled study by Guslandi et al. [54], *S. boulardii* reduced the clinical index score and achieved clinical remission, which was confirmed by sigmoidoscopy. Although all these studies observed a beneficial effect, more robust studies are required to provide more definitive evidence of the effectiveness of these probiotics for the treatment of active UC.

#### 3.1.2 Maintenance of remission in UC

Studies that investigated the maintenance of remission in UC and evaluated clinical outcomes are summarized in Table 2.

**Table 2:** Probiotics in UC for the maintenance of remission

**Probiotic**	**Author/year of publication/country**	**Study design**	**Methods**	**Results**	**Summary**
Bifidobacteria-fermented milk	Ishikawa et al. 2003 [55]	RCT	**Patients:** diagnosed with UC at least 1 year previously	Success in maintaining remission and preventive effects on the relapse of UC	A beneficial effect was observed
			**Treatment period:** 1 year		
			**T(** ***n*** **= 11):** Bifidobacteria-fermented milk product (which contained at least 10^10^ per 100 mL bottle) 100 mL each day		
	Japan				
			**C(** ***n*** **= 10):** none		
Bifidobacteria-fermented milk	Matsuoka et al. 2018 [56]	DB, RPCT	**Patients:** quiescent UC	No differences were observed between the BFM and placebo groups	The study was discontinued for lack of efficacy
			**Treatment period:** 48 weeks		
	Japan		**T(** ***n*** **= 98):** Bifidobacteria-fermented milk (*B. breve* strain Yakult (10^10^ bacteria) and *L. acidophilus* (10^9^ bacteria) )100 mL/day		
			**C(** ***n*** **= 97):** placebo		
*E. coli* Nissle 1917	Rembacken et al. 1999 [33]	DB, DD, RCT	**Patients:** UC in remission	An equivalent effect to mesalazine in maintaining remission of UC	A beneficial effect was observed
			**Treatment period:** a maximum of 12 months		
	United Kingdom				
			**T(** ***n*** **= 44):** *E. coli* Nissle 1917 at a dose of two capsules daily (2.5 × 10^10^ viable bacteria per capsule)		
			**C(** ***n*** **= 39):** mesalazine 400 mg three times daily		
*E. coli* Nissle 1917	Kruis et al. 2004 [57]	DB, DD, RCT	**Patients:** UC in remission (CAI ≦ 4, EI ≦ 4, no signs of acute inflammation on histological examination); at least two acute attacks of UC prior to the study; a duration of the current remission of no longer than 12 months	Promising behavior in sustaining the remission phase, prevention from inflammatory state; the probiotic EcN provides significantly equivalent efficacy in preventing relapses of UC and is not inferior to the established gold standard mesalazine	A beneficial effect was observed
	Germany				
			**Treatment period:** 12 months		
			**T(** ***n*** **= 162):** 200 mg/day (2.5–25 **×** 10^9^ viable bacteria)		
			**C(** ***n*** **= 165):** mesalazine		
*E. coli* Nissle 1917	Kruis et al. 1997 [58]	DB, DD, RCT	**Patients:** UC in remission	An equivalent effect to mesalazine in maintaining remission of UC	A beneficial effect was observed
			**Treatment period:** 12 weeks		
			**T(** ***n*** **= 50):** 200 mg/day (day 1–4, only 100 mg/day) of Mutaflor (100 mg contains 2.5 × 10^10^ viable *E. coli* Nissle 1917)		
	Germany				
			**C(** ***n*** **= 53):** mesalazine		
*E. coli* Nissle 1917	Henker et al. 2008 [59]	RCT	**Patients: c**hildren aged between 11 and 18; in remission for at least 3 months	An equivalent effect to mesalazine in maintaining remission of UC	A beneficial effect was observed
			**Treatment period:** 4 weeks		
			**T(** ***n*** **= 24):** 2 capsules of *E. coli* Nissle 1917 (2.5 × 10^10^ viable bacteria per capsule) while tapering off mesalazine		
	Germany				
			**C(** ***n*** **= 10):** mesalazine		
VSL#3	Venturi et al. 1999 [60]	OL	**Patients:** UC in remission; intolerant or allergic to mesalazine	Increase in fecal concentrations of *S. salivarius* ssp. *thermophilus*, lactobacilli, and bifidobacterial; maintaining the remission	A beneficial effect was observed
			**Treatment period:** 12 months		
			**T(** ***n*** **= 20):** VSL#3 (containing 5 × 10^11^ cells/g) 2 doses of 3 g		
	Italy				
			**No control group**		
VSL#3	Miele et al. 2009 [46]	DB, RPCT	**Patients:** mild-to-moderate active UC; children age between 1.7 and 16.1	Maintained remission	A beneficial effect was observed
			**Treatment period:** 1 year		
	Italy		**T(** ***n*** **= 14):** VSL#3 4.5 × 10^11^ – 1.8 × 10^12^ (based on weight) bacteria/day; concomitant steroid induction and mesalamine		
			**C(** ***n*** **= 15):** placebo; mesalazine		
*Lactobacillus* GG	Zocco et al. 2006 [61]	RCT	**Patients:** UC	Clinical remission; more effective than mesalazine in prolonging the relapse-free time	A beneficial effect was observed
			**Treatment period:** 12 months		
			**T1(** ***n*** **= 65):** *Lactobacillus* GG 18 × 10^9^ viable bacteria/day		
			**T2(** ***n*** **= 62):** *Lactobacillus* GG 18 × 10^9^ viable bacteria/day; mesalazine 2,400 mg/day		
	Italy				
			**C(** ***n*** **= 60):** mesalazine 2,400 mg/day		
*L. acidophilus* La-5 and *Bifidobacterium animalis* subsp. *lactis* BB-12 (Probio-Tec AB-25)	Wildt et al. 2011 [63]	DB, RPCT	**Patients:** left-sided UC colitis in remission	No effect on maintenance of remission in patients with UC could be demonstrated; no significant clinical benefit concerning a number of days until relapse could be demonstrated	No benefit was observed
			**Treatment period:** 52 weeks		
			**T(** ***n*** **= 20):** Probio-Tec AB-25 1.5 × 10^11^ CFU daily (two capsules three times daily)		
	Denmark				
			**C(** ***n*** **= 12):** placebo		
*Lactobacillus salivarius* subsp. s*alivarius* UCC118 or *B. infantis* 35624	Shanahan et al. 2006 [64]	DB, RPCT	**Patients:** remission of UC	No difference in relapse time between probiotics and placebo	No benefit was observed
			**Treatment period:** 1 year		
			**T(** ***n*** **= 52-53):** 10^9^ probiotics daily		
			**C(** ***n*** **= 52-53):** placebo		
	NC				
Bio-Three	Yoshimatsu et al. 2015 [62]	DB, RPCT	**Patients:** UC in remission	Lower relapse rates than placebo	A beneficial effect was observed
			**Treatment period:** 12 months		
			**T(** ***n*** **= 23):** 9 Bio-Three tablets/day		
			**C(** ***n*** **= 23):** placebo		
	Japan				
Multi-strain probiotic	Bjarnason et al. 2019 [66]	DB, RPCT	**Patients:** stabile UC in remission	Reduce the fecal calprotectin levels	A beneficial effect was observed
			**Treatment period:** 4 weeks		
			**T(** ***n*** **= 40):** 1 mL/kg Symprove (*L. rhamnosus* NCIMB 30174, *L. plantarum* NCIMB 30173, *L. acidophilus* NCIMB 30175, and *Enterococcus faecium* NCIMB 30176) each morning on a fasting stomach		
	United Kingdom				
			**C(** ***n*** **= 41):** placebo		
Biotop capsule	Lee et al. 2022 [65]	OS	**Patients:** UC whose IBS-like symptoms persisted during endoscopic remission	Improvement of bowel-related symptoms and quality of life	A beneficial effect was observed
			**Treatment period:** 1 month		
			**T(** ***n*** **= 43):** Biotop capsule (*L. acidophilus*, 75 mg; *C. butyricum* TO-A, 25 mg; *B. mesentericus* TO-A, 25 mg; and *S. faecalis* T-110, 5 mg) 3 times daily		
	Korea				

OL: open label; DB: double blind; DD: double dummy; RPCT: randomized placebo-controlled trial; RCT: randomized controlled trial; OS: observational study; C: control group; T: treatment group; NC: not clear.

##### 3.1.2.1 Bifidobacterium

Bifidobacteria-fermented milk was proven to be effective in maintaining remission and preventing the relapse of UC by Ishikawa et al. [55]. However, Matsuoka et al. [56] did not corroborate this conclusion. The trial was stopped because there were no differences in preventing relapse or Sutherland UCDAI scale scores between the Bifidobacteria-fermented milk and placebo arms. The authors opined that the lack of effectiveness may be attributable to the mode of delivery or dose of *Bifidobacterium* species, rather than to the lack of efficacy of the bacterial culture itself. Due to the lack of endoscopic analysis in the trial, the effect of Bifidobacteria-fermented milk on patient mucosal status could not be determined.

##### 3.1.2.2 *E. coli* Nissle 1917

Three large RCTs compared *E. coli* Nissle 1917 with mesalazine [33,57,58]. No significant differences were found between the two interventions with respect to relapse rate or side effects. One pediatric trial [59] also confirmed this conclusion. *E. coli* Nissle 1917 was rated as grade A recommendations [50] for maintaining UC remission, as it showed a similar effect to mesalazine and with good safety and tolerance profile.

##### 3.1.2.3 VSL#3

In an open-label study by Venturi et al. [60], VSL#3 was found to increase the fecal concentrations of *Streptococcus salivarius* ssp. *Thermophilus*, lactobacilli, and bifidobacterial in remission patients. Another study also demonstrated the role of VSL#3 in inducing and maintaining remission in active UC [46]. VSL#3 was recommended as grade A for the maintenance of UC remission by the fourth Triennial Yale/Harvard Workshop [50].

##### 3.1.2.4 Other probiotics

Several clinical trials have investigated *Lactobacillus*, Bio-Three, and multi-strain probiotics. Zocco et al. [61] enrolled 187 patients and found *Lactobacillus* GG had an equivalent effect to mesalazine in terms of relapse rate but was more effective than mesalazine in terms of lengthening relapse-free duration. In the study by Yoshimatsu et al. [62], Bio-Three arm had a lower relapse rate than the placebo arm. Two RCTs [63,64] found no difference in UC remission maintenance between *Lactobacillus* plus *Bifidobacterium* and placebo, but an observational study found that a combination of *L. acidophilus*, *Clostridium butyricum*, *Bacillus mesentericus*, and *Streptococcus faecalis* [65]. Bjarnason et al. [66] found that multi-strain probiotic was better than placebo in reducing the fecal calprotectin levels, but there were no significant differences with respect to IBD quality of life questionnaire scores between the two groups. These studies involving probiotics outside the mainstream provide only flimsy evidence of their beneficial role in maintaining remission of UC.

### 3.2 Probiotics in CD

#### 3.2.1 Induction of remission in active CD

Few studies have investigated the use of probiotics in patients with active CD. Table 3 presents a summary of the clinical outcomes of these studies.

**Table 3:** Probiotics in CD for the induction of remission

Probiotic	Author/year of publication/country	Study design	Methods	Results	Summary
*Lactobacillus* GG	Gupta et al. 2000 [67]	OL	**Patients:** 4 male children with mild-to-moderately active CD	Improvement in clinical activity, intestinal permeability, intestinal barrier function, and clinical status; decrease in CDAI score	A beneficial effect was observed
			**Treatment period:**6 months		
			**T(** ***n*** **= 4):** *Lactobacillus* GG (10^10^ colony-forming units [CFU]) in enterocoated tablets twice a day		
	United States				
			**No control group**		
*Lactobacillus* GG (L. GG)	Schultz et al. 2004 [68]	DB, RPCT	**Patients**: moderate-to-active CD	No differences were observed between the L. GG and placebo groups. The benefit of L. GG in inducing or maintaining medically induced remission in CD was not demonstrated	No benefit was observed
			**Treatment period**: 6 months		
			**T(** ***n*** **= 5)**: L. GG (2 × 10^9^ CFU/day; CAG Functional Foods, Omaha, NE)		
	United States				
			**C(** ***n*** **= 6)**: placebo		
Combined probiotics (*Bifidobacterium* and *Lactobacillus*)	Fujimori et al. 2007 [69]	OL	**Patients**: active CD patients without history of operation for CD	Decrease in CDAI, IOIBD scores, incidence of daily diarrhea, and index of abdominal pain	A beneficial effect was observed
			**Treatment period**: 13.0 ± 4.5 months		
	Japan		**T(** ***n*** **= 10)**: a synbiotic therapy consisting of probiotics (7.5 × 10^10^ colony forming units CFU per day, including *Bifidobacteria* and *Lactobacillus*) and probiotics Yuan (9.9 g per day)		
			**No control group**		
*E. coli* Nissle 1917	Malchow 1997 [71]	DB, RPCT	**Patients**: active colonic CD	The use of *E. coli* strain Nissle 1917 as an adjuvant for patients with colonic CD can reduce the intake of prednisolone and stop using steroids during the trial	A beneficial effect was observed
			**Treatment period**: 1 year		
	Germany		**T(** ***n*** **= 16)**: capsules containing a preparation of viable, nonpathogenic *E. coli* Nissle 1917		
			**C(** ***n*** **= 12)**: placebo		
Synbiotic comprising *B. longum* and Synergy 1	Steed et al. 2010 [70]	DB, RPCT	**Patients**: active CD	Decrease in CDAIs, histological scores and TNF-a expression; mucosal bifidobacteria proliferated	A beneficial effect was observed
			**Treatment period**: 6 months		
			**T(** ***n*** **= 13)**: 2 × 10^11^ freeze-dried viable *B. longum* in a gelatin capsule and a sachet containing 6 g of Synergy I twice daily		
	United Kingdom				
			**C(** ***n*** **= 11)**: placebo		

OL: open label; DB: double blind; RPCT: randomized placebo-controlled trial; RCT: randomized controlled trial; C: control group; T: treatment group.

##### 3.2.1.1 *Lactobacillus* GG

Two studies investigated the effect of *Lactobacillu*s in patients with active CD. Gupta et al. [67] discovered that after taking enteric-coated pills containing *Lactobacillus* GG for 1 week, four children with mild-to-moderate CD showed significant improvement in clinical activity and continued to improve throughout 24 weeks. The median CD activity index (CDAI) score of children was 73% lower than baseline, and the intestinal permeability was also improved. Subsequently, Schultz et al. [68] conducted a double-blind RCT to investigate whether oral *Lactobacillus* GG can induce or maintain remission in CD. However, only five patients (5/11) completed the study, with two remissions in both the intervention and control groups and no significant between-group difference.

##### 3.2.1.2 Other probiotics

Several other studies with different probiotic strains have been performed and the results are summarized in Table 3. In a single-arm trial by Fujimori et al. [69], high-dose combined-probiotics (*Bifidobacterium* and *Lactobacillus*) mixed with prebiotics (psyllium) (mean duration of intervention: 13.0 ± 4.5 months) were found to be safe and beneficial in the treatment of active CD. After treatment, seven of ten patients showed improvement in clinical symptoms and a significant decrease in the CDAI and International Organization for the Study of IBD (IOIBD) scores; two of these patients were able to stop using prednisolone, while four others reduced their dosage. In another placebo-controlled trial, CD patients treated with Synbiotic (comprised of *B. longum* and Synergy 1) showed a significant decrease in CDAI and histological scores, and improved proliferation of *Bifidobacterium* mucosae [70]. Malchow [71] found that use of *E. coli* strain Nissle 1917 as an adjuvant may help reduce the prednisolone dose in individuals with colonic CD. Despite these demonstrated benefits of probiotic strains in CD, the quality and the number of studies are limited. More placebo-controlled trials are required to provide robust evidence.

#### 3.2.2 Maintenance of remission in CD

Studies that investigated the use of probiotics in the maintenance of remission in CD and the evaluated clinical outcomes are summarized in Table 4.

**Table 4:** Probiotics in CD for the maintenance of remission

Probiotic	Author/year of publication/country	Study design	Methods	Results	Summary
*S. boulardii*	Garcia et al. 2008 [72]	RPCT	**Patients:** CD in remission	The intestinal permeability has been improved, the lactulose/mannitol ratio has decreased, and the barrier function has improved; even though complete normalization was not achieved	A beneficial effect was observed
			**Treatment period**:3 months		
			**T(** ***n*** **= 14)**: *S. boulardii* every 8 h as an oral capsule formulation which contained 200 mg lyophilized *S. boulardii*-17 (about 4 × 10^8^ cells), 6 mg sucrose, and 2.4 mg magnesium stearate		
	Brazil				
			**C(** ***n*** **= 17)**: placebo every 8 h as an oral capsule containing 200 mg cellulose, 6 mg sucrose, and 2.4 mg magnesium stearate		
*S. boulardii*	Guslandi et al. 2000 [73]	RCT	**Patients**: CD in clinical remission	The clinical recurrence of CD in patients receiving mesalazide plus Bulazyme maintenance treatment was significantly reduced	A beneficial effect was observed
			**Treatment period**: 6 months		
			**T(** ***n*** **= 16)**: *S. boulardii* 500 mg two capsules in the morning; Pentasa 500 mg two capsules twice a day		
	Italy				
			**C(** ***n*** **= 16)**: mesalazine 500 mg in a sustained-release preparation in ethylcellulose microgranules, two capsules three times a day		
*S. boulardii*	Bourreille et al. 2013 [74]	DB, RPCT	**Patients**: remission after treatment with steroids or salicylates	There were no significant differences between the groups in terms of the average activity index score of CD, the red blood cell sedimentation rate or the median level of CRP, and the median time to relapse	No benefit was observed
			**Treatment period**: 52 weeks		
	France				
			**T(** ***n*** **= 80)**: *S. boulardii* 1 g/day		
			**C(** ***n*** **= 79)**: placebo		
*L. rhamnosus strain* GG	Bousvaros et al. 2005 [75]	RPCT	**Patients**: children age between 5 and 21; CD in remission	The recurrence time and the proportion of patients in the LGG and placebo groups are basically the same. No reliable correlations were found between drug intake, clinical status, and lactic acid bacteria colonization in feces	No benefit was observed
			**Treatment period**: 2 years		
	United States				
			**T(** ***n*** **= 39)**: *L. rhamnosus strain* GG 1 capsule (containing at least 10^10^ bacteria and 295 mg inulin) twice a day		
			**C(** ***n*** **= 36)**: placebo		
*L. johnsonii*	Van Gossum et al. 2007 [76]	DB, RPCT	**Patients**: CD prior to elective ileo-cecal resection	Oral probiotic LA1 has no protective effect on early endoscopic recurrence of CD patients undergoing ileocecal resection. The histological scores, serum inflammatory indexes, and clinical recurrence rates of the two groups were similar	No benefit was observed
			**Treatment period**:12 weeks		
			**T(** ***n*** **= 34)**: *L. johnsonii* (LA1, Nestec) in freeze-dried form and blended with maltodextrin at 10^10^ colony-forming units (CFU)/day		
			**C(** ***n*** **= 36)**: placebo		
	Belgium				
*L. johnsonii*	Marteau et al. 2006 [77]	DB, RPCT	**Patients**: patients had undergone surgical resection of <1 m, removing all macroscopic lesions within the past 21 days	Endoscopic score distribution did not differ significantly between the LA1 and placebo groups. *L. johnsonii* LA1 (4 × 10^9^ CFU/day) did not have a sufficient effect, if any, to prevent endoscopic recurrence of CD	No benefit was observed
			**Treatment period**: 6 months		
	France				
			**T(** ***n*** **= 48)**: two packets per day of lyophilized LA1 (2 × 10^9^ CFU; ***n*** **=** 48)		
			**C(** ***n*** **= 50)**: placebo		
*Lactobacillus* GG	Prantera et al. 2002 [78]	DB, RPCT	**Patients**: patients operated on for CD in whom all of the diseased gut had been removed	*Lactobacillus* GG seems neither to prevent endoscopic recurrence at 1 year nor reduce the severity of recurrent lesions	No benefit was observed
			**Treatment period**: 52 weeks		
			**T(** ***n*** **= 23)**: 2.46 g bags containing *Lactobacillus* GG (Dicoflor 60; Dicofarm, Rome, Italy) 6 × 10^9^ colony forming units (cfu) twice daily		
	Italy				
			**C(** ***n*** **= 22)**: placebo		
*Synbiotic* 2000	Chermesh et al. 2007 [81]	DB, RPCT	**Patients**: CD undergoing resection	No differences were observed between the 2 groups in the clinical, laboratory, and endoscopic outcome	No benefit was observed
			**Treatment period**:1 year		
			**T(** ***n*** **= 20)**: *Synbiotic* 2000 (contains prebiotics and probiotics, including 10^10^ *Pediacoccus pentoseceus*, 10^10^ *L. raffinolactis*, 10^10^ *L. paracasei* subsp. *paracasei* 19, 10^10^ *L. plantarum* 2,362; and 2.5 g *β*-glucans, 2.5 g inulin, 2.5 g pectin, and 2.5 g resistant starch) once daily		
	Israel				
			**C(** ***n*** **= 10)**: placebo		
VSL#3	Madsen et al. 2008 [80]	DB, RPCT	**Patients**: CD patients who had a recent ileal-colonic resection with a small intestine to colon anastomosis	Patients receiving VSL#3 had significantly reduced levels of ileal mucosal pro-inflammatory cytokines, IL-1β, TNFα, and IFN-γ, and increased levels of the immunomodulatory cytokine, TGFβ (*p* < 0.05), compared to patients receiving placebo, and less severe endoscopic recurrence (Rutgeerts Grades 3 or 4)	A beneficial effect was observed
			**Treatment period**: 90 days		
			**T(** ***n*** **= 58)**: one sachet of VSL#3 (9 × 10^11^ bacteria) twice daily		
	NC				
			**C(** ***n*** **= 62)**: placebo		
*Multi-strain* probiotic	Bjarnason et al. 2019 [66]	DB, RPCT	**Patients**: stabile CD in remission	No significant changes were seen in CD	No benefit was observed
			**Treatment period**:4 weeks		
			**T(** ***n*** **= 33)**: treatment with *Multi-strain* probiotic 1 mL/kg/day		
	United Kingdom				
			**C(** ***n*** **= 29)**: placebo		

OL: open label; DB: double blind; RPCT: randomized placebo-controlled trial; RCT: randomized controlled trial; C: control group; T: treatment group; NC: not clear.

##### 3.2.2.1 S. boulardii

There are three RCTs associated with *S. boulardii*. The duration and dose of these trials were radically different, and the final results were likewise somewhat conflicting. Garcia Villela et al. [72] discovered that compared to placebo, *S. boulardii* improved the intestinal permeability of CD patients in remission as well as decreased the lactulose/mannitol ratio and altered intestinal mucosal barrier integrity while maintaining the baseline drug treatments (mesalazine, azathioprine, prednisone, metronidazole, and/or thalidomide) unchanged. Guslandi et al. [73] reported that 32 CD patients receiving mesalamine combined with *S. boulardii* had a significantly lower 6-month clinical recurrence rate (6.25%) than patients receiving standard mesalamine therapy (37.5%). In addition, there was improvement in diarrhea, abdominal pain, overall health, CDAI, and hematocrit levels. Nevertheless, Bourreille et al. [74] reported contrary results. They found no significant differences in mean CDAI scores, median ESR, CRP levels, or median time to relapse between patients receiving *S. boulardii* or placebo. Because of the inconsistent effects of trials of the probiotic yeast *S. boulardii*, more studies are still required to assess its effectiveness and safety for patients with CD in remission.

##### 3.2.2.2 Lactobacillus

We found four studies related to *Lactobacillus*, none of which showed its effectiveness in maintaining the remission of CD. Bousvaros et al. [75] tracked 75 CD adolescents for 2 years and found that the relapse time and proportion of patients in the *Lactobacillus* intervention and placebo arms were essentially the same, with no significant differences in medication intake, clinical state, or fecal lactate. There was no strong link between bacterial colonization and health. Three other studies [76,77,78] examined the effect of *Lactobacillus* on the time to recurrence in patients after surgery, with endoscopic relapse as the primary outcome. In the study by Van Gosum et al. [76], oral *Lactobacillus johnsonii* intervention in 70 CD patients after ileocecal resection did not prevent early recurrence. Marteau et al. [77] investigated CD patients who received 2 sachets of lyophilized *L. johnsonii* (2 × 10^9^ CFU) or placebo per day for 6 months and concluded that *L. johnsonii* was ineffective in preventing endoscopic microscopic inspection of CD recurrence following CD bowel resection. Prantera et al. [78] studied the effects of oral *Lactobacillus* GG in CD patients in whom the diseased part of the gut was surgically removed for a year; they found that it did not prevent endoscopic recurrence, nor did it diminish the incidence of relapse and severity. Moreover, a meta-analysis [79] found that using *Lactobacillus* GG as a maintenance medication may increase the recurrence rate of CD when compared to placebo and that *Lactobacillus* GG was less successful in lowering the relapse rate.

##### 3.2.2.3 Other probiotics

A small number of clinical trials have also investigated the effects of Synbiotic 2000, VSL#3, and multi-strain probiotics, most of which were on post-operative patients. Patients receiving VSL#3 showed significantly lower levels of ileal mucosal pro-inflammatory cytokines IL-1β, TNF-α, and IFN-γ, higher levels of TGF-β, and milder endoscopic recurrence than placebo [80]. Chermesh et al. [81] reported that daily administration of Synbiotic 2000 (a cocktail rich in four probiotics and four prebiotics) showed no effect on postoperative recurrence in CD patients. Multiple strains of probiotics that reduced intestinal inflammation in UC patients showed no therapeutic effect on CD [66]. More research is needed to evaluate whether these probiotics can lower the occurrence of clinical relapse.

Overall, based on the available results, *E. coli* Nissle 1917 may be available for maintenance treatment of UC in remission; in addition, *Bifidobacterium* and VSL#3 may be available for the induction of remission in mild to moderately active UC. Definitive conclusions on the effectiveness of probiotics in CD cannot yet be formed. All clinical trials have shown the effectiveness of *E. coli* Nissle 1917 as a maintenance treatment for UC in remission. In addition, both *Bifidobacterium* and VSL#3 have been shown to induce remission in mild-to-moderately active UC. However, there is inconsistent/insufficient evidence of the effectiveness of other probiotics in inducing or maintaining remission of UC. Similarly, clinical trials of probiotics for CD have demonstrated their ineffectiveness or yielded inconsistent results.

## 4 Mechanism of probiotics in IBD

**Figure 1:**
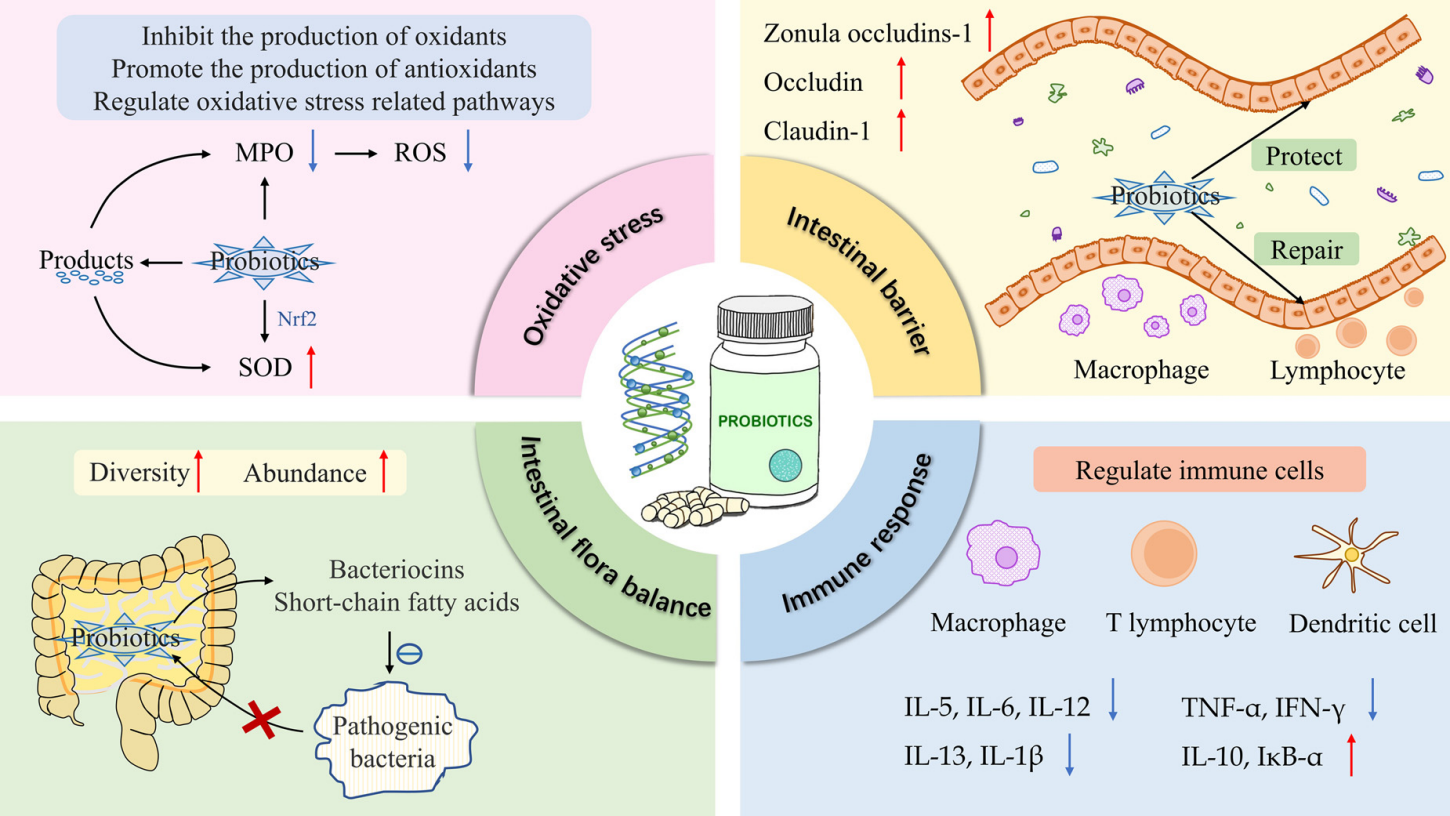
Schematic illustration of the mechanism of action of probiotics in IBD.

Probiotics alleviate or treat IBD by mitigating oxidative stress, repairing the intestinal barrier, regulating intestinal flora balance, and modulating intestinal immune response. A schematic illustration of their mechanism of action is shown in Figure 1.

### 4.1 Mitigation of oxidative stress

Studies have shown that oxidative stress plays a key role in the development of IBD and that the imbalance between reactive oxygen species (ROS) accumulation and antioxidant activity is closely related to the incidence and severity of IBD [82]. Low-to-moderate concentrations of ROS are associated with the maintenance of normal intestinal homeostasis; however, intestinal inflammation leads to the production of excessive ROS, resulting in damage to cell structure and function and increased intestinal permeability, further aggravating inflammation [83]. MPO, which is specific to neutrophils and monocytes, is an abundant granulocyte heme enzyme. ROS can be efficiently produced by MPO through halogenation or peroxidase cycle. *Lactobacillus plantarum* supplementation and *E. coli* Nissle 1917 can reduce MPO levels to optimize the immune barrier [84,85]. Superoxide dismutase (SOD) is an important antioxidant enzyme and a prime scavenger of oxidative free radicals in the body. The severity of IBD is related to the level of SOD. *Bifidobacterium quadruplex* combined with mesalazine for UC can increase the level of SOD [86]. *L. plantarum* and *Bifidobacterium* activate the Nrf2 pathway at the transcriptional level and upregulate antioxidant factors (e.g., SOD1, SOD2, GPX2), with significant effects on DSS-induced UC in mice [87]. The product of probiotics can also modulate oxidative stress-related pathways. Γ-Glutamylcysteine, an antioxidant secreted by *Bifidobacterium*, can inhibit endoplasmic reticulum stress-mediated ROS [88]. The tryptophan metabolite indole-3-lactic acid produced by *B. infantis* metabolism induces increased mRNA expression of SOD2 and NAD(P)H dehydrogenase [89]. In summary, probiotics can reduce the intestinal inflammatory response by inhibiting the production of oxidants, promoting the production of antioxidants, and regulating oxidative stress-related pathways.

### 4.2 Repair of intestinal barrier

The intestinal mucosal barrier is the first line of defense in the intestine against bacteria and viruses, which can be divided into four parts: mechanical barrier, microbial barrier, chemical barrier, and immune barrier [90]. IBD is characterized by varying degrees of intestinal mucosal injury, and studies have shown that probiotics can improve the damaged mucosal epithelial barrier. The tight junctions surrounding the apical side of the intestinal epithelium are the structural basis for maintaining the mechanical barrier. *E. coli* Nissle 1917 was shown to increase the expression of zonula occludins-1 in mouse intestinal epithelial cells and provide protection against the increased permeability of luminal material in the mucosa-associated with DSS colitis [91]. *B. longum* and VSL#3 gavage were shown to increase the expression of zonula occludins-1, ocludin, and claudin-1 in the colon tissue in a mouse model of TNBS-induced colitis [92]. The intestinal mucus layer in the outermost layer of the intestinal mucosal barrier is the first line of defense against harmful substances and pathogens in contact with the intestinal internal environment and is also a major part of the chemical barrier. In a study, *Lactobacillus rhamnosus* CNCM I-3690 physically maintained the regulated phagocytes and mucus layer, while counteracting changes in local and systemic lymphocytes [93]. Several genes involved in mucus layer production, including *Muc2* (LFC 2.2), *Muc6* (LFC 3.7), *Muc5b* (LFC 2.9), and *Muc4* (LFC 1.24), were significantly upregulated in the *B.* breve UCC2003 experimental group [94]. VSL#3 inhibits pro-inflammatory chemokine KC, monocyte chemoattractant protein-1, and macrophage inflammatory protein-2 and upregulates tissue regenerative growth factor transforming growth factor-β, fibroblast growth factor-1, and vascular endothelial growth factor-A, resulting in accelerated relief of colitis symptoms in Muc2-deficient mice [95]. Overall, probiotics repair the intestinal barrier by repairing the mucosal epithelium and promoting the production of the Intestinal mucus layer.

### 4.3 Regulation of intestinal flora balance

Probiotics, which are indispensable for human health, play an important role in regulating intestinal flora. Probiotics are involved in synthesizing various vitamins, digesting food, promoting intestinal peristalsis, inhibiting the growth of pathogenic flora, and decomposing harmful and toxic substances [96]. Probiotics can inhibit the growth of pathogenic bacteria by producing bacteriocins and short-chain fatty acids and inhibit the multiplication and invasion of pathogenic bacteria by competing with pathogenic bacteria for nutrients [97]. Probiotics form a microbial barrier after they colonize the intestine, preventing pathogenic bacteria from adhering to and invading the intestine [98]. Selenium-enriched *B. longum* DD98 was found to improve the diversity of UC intestinal flora and promote the abundance of beneficial bacteria, including Lachnospiraceae, Lactobacillaceae, and Prevotellaceae at family level [99]. Compared to DSS-induced colitis mice, gavaged *L. rhamnosus* GG mice had higher relative abundance of two clades, Firmicutes and Bacteroidetes, and lower relative abundance of *Proteobacteria* and *Epsilonbacteraeota*. At the genus/species level, groups of Muribaculacea, Rikenellaceae_RC9_gut_group, and Lachnospiraceae_NK4A136_group were reduced, while *Helicobacter* and *Escherichia–Shigella* were significantly increased in mice with DSS-induced colitis [100]. In different chemical-induced IBD mice models, chitosan/sodium alginate-coated *E. coli* Nissle 1917 alleviated inflammation, repaired the colonic epithelial barrier, modulated the intestinal microbial community, and improved the abundance of *Lachnospiraceae*_NK4A136 and *Odoribacter* in the intestinal flora [101]. The formation of a flora of beneficial bacteria in the intestinal tract helps maintain the balance of intestinal flora and stops the onset and development of IBD.

### 4.4 Regulation of intestinal immune response

IBD is a chronic immune-mediated inflammatory disease. Probiotics and their metabolites can activate innate immunity and induce adaptive immunity to regulate immune cells (such as macrophages, T lymphocytes, DCs), bind Toll-like receptors, and activate signaling pathways, such as NF-κB, JAK/STAT, and MAPK [102,103,104]. IBD inflammation can be reduced by modulating the immune response, reducing the production of inflammatory factors, and promoting the secretion of anti-inflammatory factors. Treatment of colitis with *E. coli* Nissle 1917 resulted in restoration of secretory immunoglobulin A levels and reduction of IL-5, IL-13, TNF-α, and IFN-γ levels [105]. *Lactobacillus* spp. downregulated *JAK*, *TIRAP*, *IRAK4*, *NEMO*, and *RIP* genes in the NF-κB pathway, with different *STAT* gene expressions, as well as reduced IL-6 and IL-1β production [106]. *L. plantarum* ZS62 downregulated the serum levels of IL-1β, IL-6, IL-12, TNF-α, and IFN-γ, and the relative mRNA and protein expression of IL-1β, IL-12, and TNF-α in colonic tissues of IBD mice, with upregulation of serum and relative mRNA and protein expression levels of IL-10 [107]. Probiotics can alleviate IBD symptoms by reducing the degree of intestinal inflammation in the gut.

## 5 Discussion and conclusions

Despite the availability of a diverse range of biological agents and molecular-targeted therapies, primary and subsequent treatment failure rates for IBD continue to be high. Thus, development of novel therapeutic targets and calibration of the existing therapies are key imperative to improve the effectiveness, safety, and tolerability [108]. Despite the relative safety of probiotics, some patients tend to perceive these as health supplements rather than a treatment. Probiotics with demonstrable efficacy have great potential to move from supplemental to therapeutic agents for IBD in the future. Indeed, probiotics should be used more often as an alternative or as a supplement to conventional treatment in patients with IBD [109].

All trials have demonstrated the benefits of *Bifidobacterium* and VSL#3 in inducing remission of mild-to-moderate active UC with no side effects. Moreover, the efficacy of *E. coli* Nissle 1917 in maintaining UC remission has been found to be comparable to that of mesalazine. These are consistent with the positive results of three meta-analyses [110,111,112]. Moreover, probiotics may play a role in promoting mucosal healing, as mucosal performance assessment has become a standard part of IBD trials [113]. However, clinical trials investigating the effectiveness of other probiotics in the treatment of UC have yielded contradictory findings. The effect of probiotics on CD has been disappointing so far, with most studies showing ineffectiveness and conflicting evidence; thus, there is a lack of sufficient evidence to recommend their usage [110,114,115].

Available evidence suggests that probiotics are effective in relieving or treating IBD by alleviating oxidative stress, repairing the intestinal barrier, regulating the balance of intestinal flora, and modulating the intestinal immune response. The disparities in the benefits of probiotics between CD and UC may reflect the complexities of the probiotic (bacteria)–host interactions. The lesions of UC involve the mucosal and submucosal layers, whereas CD typically involves the entire intestinal wall, which may make intestinal repair more difficult in CD than in UC. Activation of the intestinal mucosal response is the direct cause of the onset and development of intestinal inflammation in IBD, where the most important cells are Th (Th1, Th2, Th17) and Tregs. CD is mediated by Th1, while UC is mediated by Th2. *B. infantis* was shown to promote Th1 and suppress Th2 immune responses [116]. *Lactobacillus fermentum* resulted in decreased levels of Th1, Th2, and Th17-related cytokines and increased IL-10 in the colon [117]. *Bifidobacterium* improved Th1/Th2 balance in mice, increased Th1 cytokine levels, and decreased Th2 cytokine levels in splenocytes [118]. VSL#3 retargeted allergen-specific Th2-polarized immune responses to Th1-T regulatory responses [119]. This may be the reason why probiotics have no significant relief and limited therapeutic effect in CD but are effective in UC. Compared to 24 kinds of probiotics, *L. rhamnosus* has the best effect in relieving weight loss and improving the Shannon index in the UC model; *Lactobacillus reuteri* has the best effect in reducing the UCDAI; *L. acidophilus* has the best effect in increasing the expression of tight junction protein ZO-1; and *Lactobacillus coryniformis* has the best effect in reducing the content of serum pro-inflammatory factor TNF-α [120]. Even with the same probiotic, different subtypes can show different effects, so the conditions they can treat may differ.

However, there are some limitations in these trials. First, many of these trials have not been sufficiently evaluated in terms of effectiveness, dose, or duration of administration. Second, the small sample size in these trials limits the generalizability of the findings. Third, most studies have adopted specific doses of probiotics without investigating the link between dose and response, making it difficult to compare results even for the same strains. Fourth, nearly all trials examining the effectiveness of *Bifidobacterium* in the treatment of IBD were conducted in Japan. Trials of *E. coli* Nissle 1917 for the maintenance treatment of UC in remission were conducted in Germany, while most of the trials examining VSL#3 were conducted in Italy. Regional heterogeneity may lead to bias in the effectiveness of various types of probiotics. Subgroup analyses of probiotic efficacy by geography, age, and gender were not performed in the available studies. Fifth, the western diet and its components affect the abundance, colonization, and phenotypic behavior of *E. coli* in the gut, which may trigger or contribute to intestinal inflammation. In contrast, the Mediterranean diet and specific dietary fibers can eliminate these effects and prevent inflammation [121]. The impact of diet on IBD pathogenesis and interactions with probiotics should be considered when studying the effectiveness of probiotics.

In the future, more RCTs are needed to investigate and validate the efficacy of single probiotic strains and combined probiotic applications for IBD. For probiotics with definite effects, it is recommended to investigate the bare minimum or precise probiotic required for specific advantages, which would help normalize the treatment [122]. For probiotics with unclear effects, it is recommended to investigate them from a mechanistic point of view and compare the differences in their effects to identify subsets and characteristics of IBD populations in whom probiotics are not treatment options. Use of probiotics as a preventive measure in individuals who are prone to IBD should be considered and the mechanism and course of action of probiotics should also be further understood. Moreover, probiotic engineering may be a promising new technology for the future treatment of IBD. Probiotic engineering uses suitable bacterial strains, such as *L. rhamnosus* [123], to form robust probiotic strains with enhanced functional properties that not only target the control of gut pathogenic microorganisms but also provide specific interventions for IBD [124]. Covalent-organic-framework-based artificial probiotics have been invented to treat IBD by regulating intestinal flora, suppressing intestinal inflammation, protecting intestinal epithelial cells, and modulating immunity [125]. New probiotic delivery systems are also being developed that may protect probiotics from harsh gastrointestinal conditions, improve intestinal adhesion and reduce immunogenicity [126]. Probiotics should adhere to strict guidelines from manufacturing to storage to distribution, making potential health benefits be maximized, and consumer faith in these helpful microbes can be bolstered by adopting thorough quality management measures to ensure their safety, efficacy, and consistency [127]. In addition to oral administration, fecal microbiota transplantation may be a reliable option for future treatment to improve the condition of IBD patients [128]. Fecal microbiota transplantation has been proven to be a therapeutic intervention for inducing clinical remission in UC, but achieving endoscopic remission and maintaining long-term remission remains a challenge, and there are safety concerns [129].

In conclusion, based on the available results, the use of *E. coli* Nissle 1917 for the maintenance treatment of UC in remission, and *Bifidobacterium* and VSL#3 for induction of remission of mild-to-moderately active UC is feasible. However, there is no definitive evidence of the effectiveness of other probiotics for the treatment of UC or probiotics for the treatment of CD. The mechanism of the therapeutic effect of probiotics in IBD may include reduced oxidative stress, repair of intestinal barrier, regulation of intestinal flora balance, and modulation of intestinal immune response. Differences in the benefits of probiotics between CD and UC may be attributable to the different lesion extent and immune-mediated pathophysiology in the two conditions.
